# Sarcoidosis-related autoimmune inflammation in COVID-19 convalescent patients

**DOI:** 10.3389/fmed.2023.1271198

**Published:** 2023-12-21

**Authors:** Artem Rubinstein, Igor Kudryavtsev, Annа Malkova, Jennet Mammedova, Dmitry Isakov, Irina Isakova-Sivak, Dmitry Kudlay, Anna Starshinova

**Affiliations:** ^1^Almazov National Medical Research Centre, Saint Petersburg, Russia; ^2^Institution of Experimental Medicine, Saint Petersburg, Russia; ^3^Far Eastern Federal University, Vladivostok, Russia; ^4^Ariel University Faculty of Natural Sciences, Ariel, Israel; ^5^First Saint Petersburg State I. Pavlov Medical University, Saint Petersburg, Russia; ^6^Institute of Pharmacy, I.M. Sechenov First Moscow State Medical University (Sechenov University), Moscow, Russia; ^7^NRC Institute of Immunology, Moscow, Russia; ^8^Department of Pharmacognosy and Industrial Pharmacy, Faculty of Fundamental Medicine, Moscow, Russia

**Keywords:** autoimmunity, sarcoidosis, COVID-19, post-COVID-19 syndrome, B-cell, follicular Th, follicular Treg, autoantibodies

## Abstract

Currently, there are a large number of reports about the development of autoimmune conditions after COVID-19. Also, there have been cases of sarcoid-like granulomas in convalescents as a part of the post-COVID-19 syndrome. Since one of the etiological theories of sarcoidosis considers it to be an autoimmune disease, we decided to study changes in the adaptive humoral immune response in sarcoidosis and SARS-CoV-2 infection and to find out whether COVID-19 can provoke the development of sarcoidosis. This review discusses histological changes in lymphoid organs in sarcoidosis and COVID-19, changes in B cell subpopulations, T-follicular helper cells (Tfh), and T-follicular regulatory cells (Tfr), and analyzes various autoantibodies detected in these pathologies. Based on the data studied, we concluded that SARS-CoV-2 infection may cause the development of autoimmune pathologies, in particular contributing to the onset of sarcoidosis in convalescents.

## 1 Introduction

**Figure 1:**
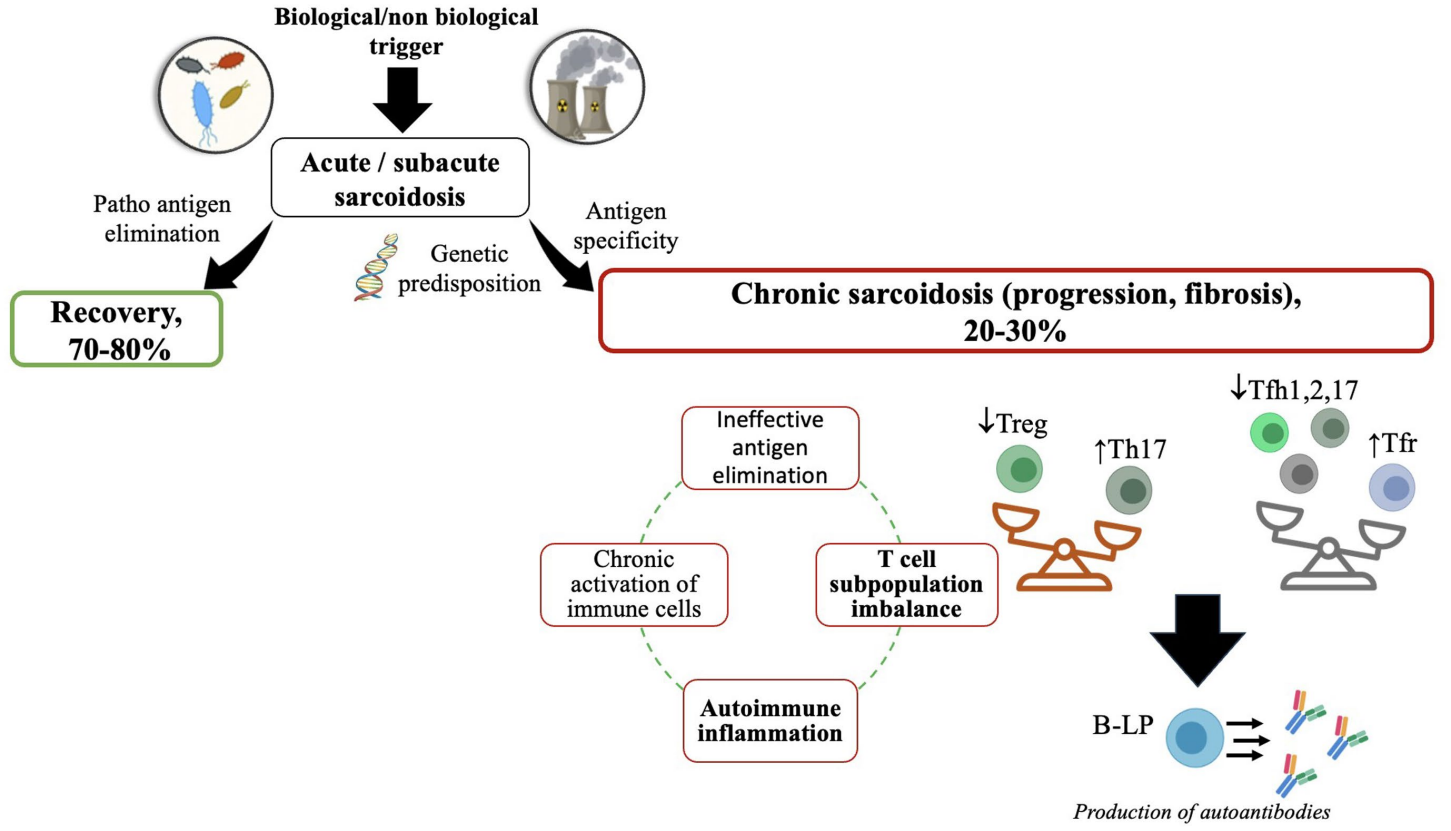
A putative scheme of the development of sarcoidosis. ↑—high level; ↓—low level. The figure was drawn by the authors.

Sarcoidosis remains to be recognized as one of the granulomatous diseases of unknown etiology (1). Multiple conducted studies confirm one of the most common theories regarding autoimmune pathogenesis behind the emergence of granulomatous inflammation that might result from bacterial and viral agents, inorganic and organic substances, vaccines, etc. (Figure 1) (2, 3). The current concept implies that caseous necrosis-free granuloma arises due to the aforementioned cues in genetically predisposed subjects, followed by the development of self-recovery or chronicity of clinical and multi-organ alterations (4–6).

Granuloma formation occurs in intrathoracic lymph nodes, lungs, skin, heart, and other organs upon contact with antigen-presenting cells (macrophages, dendritic cells, activated epithelial cells) by a triggering agent, followed by the development of unregulated autoimmune inflammation, additionally characterized by an imbalance between pro- and anti-inflammatory acquired immune cell subsets (T- and B lymphocytes) as well as regulatory T cells (7–9). Moreover, a tight link between sarcoidosis and COVID-19 caused by SARS-CoV-2 has been hypothesized, which may be another new trigger agent related to sarcoidosis, capable of either provoking or exacerbating it (10–12).

In early 2023, based on the analysis of the medical records of approximately 6 million subjects, it was shown that prior SARS-CoV-2 infection elevated the risk of developing a wide range of autoimmune diseases, including rheumatoid arthritis, ankylosing spondylitis, systemic lupus erythematosus, dermatopolymyositis, systemic sclerosis, Sjögren’s syndrome, mixed connective tissue disease, Behçet’s disease, rheumatic polymyalgia, vasculitis, psoriasis, inflammatory bowel disease, celiac disease, and type 1 diabetes (13). It is believed that genetic and environmental factors act as the major causes contributing to the development of autoimmune diseases, whereas infectious events coupled with viral, bacterial, and fungal infections may serve as one of the most crucial triggers in the emergence of immune system impairment resulting in autoimmunity (14). Moreover, mechanisms such as molecular mimicry, recognition of similar epitopes derived from protein molecules, and polyclonal activation of T- and B cells may affect virus-induced autoimmune diseases. Similarly, an important cue resulting in the development of autoimmune pathologies may be an uncontrolled inflammatory response related to the overproduction of pro-inflammatory cytokines (15), which may be closely related to a cytokine storm in severe COVID-19 and long-COVID-19 sequelae, including autoimmune reactions (16–18). In this regard, it has been reported that psoriatic arthritis (19, 20), systemic lupus erythematosus (21, 22), and other organ-specific and systemic autoimmune manifestations (23, 24) can be observed after COVID-19 infection. Moreover, 33 aberrantly expressed genes common to COVID-19 and sarcoidosis were discovered and functionally analyzed to reveal that such genes are associated with the production of cytokines involved in the immune response and T cell cytokine production (25). In addition, inflammatory aggregates consisting of macrophages, multinucleated epithelioid cells, and CD4+ T cells that histologically resembled sarcoidosis-related granulomatous events were detected during postmortem examination of lung biopsies from COVID-19 patients (26–28).

The aim of the review was to determine autoimmune features in patients with sarcoidosis and to assess immune disorders as predictors of activation and progression post-COVID-19.

## 2 Review analysis methods

We analyzed original papers and reviews covering the period from December 2019 to May 2023, published in accessible international databases (“Medline,” “PubMed,” and “Scopus”), with queries for the keywords “COVID-19,” “SARS-CoV-2,” “sarcoidosis,” “Treg,” “follicular Treg,” and “Treg subsets.” Inclusion criteria were as follows: original research with observation of patients with sarcoidosis and COVID-19, meta-analysis, reviews, and research articles; exclusion criteria: books, clinical trials, and clinical cases.

The analysis was carried out in accordance with the PRISMA protocol^1^ used for this type of study.

## 3 Onset of sarcoidosis during or after COVID-19

Granuloma formation associated with clinical cases in post-COVID-19 patients is one of the most crucial confirmations of this event after a coronavirus infection. Clinical cases accompanied by the emergence of symptoms and manifestations of sarcoidosis during or after COVID-19, in addition to post-vaccination after the SARS-CoV-2 infection, are shown in Table 1.

**Table 1:** Clinical cases of sarcoidosis onset during or after COVID-19.

Authors, year of publication	Patients (sex, age)	Onset of symptoms	Symptoms	Treatment and outcome
Behbahani et al. (29)	Woman, 72 years old	2 weeks after recovery from COVID-19 pneumonia	Cutaneous, painful, firm nodules representing noncaseating sarcoid-like granulomas	Clobetasol ointment: granulomas gradually reduced within 25 days.
Polat Ekinci (30)	Woman, 55 years old	2–3 weeks after COVID-19	Sarcoid-like granulomas mimicking cicatricial syndrome. 1–2 cm round, mobile and tender subcutaneous nodules on both arms, 3–4 mm size three subcutaneous papules in the periorbital area	No treatment was administered to the female patient due to the lack of any evidence of systemic sarcoidosis, and the lesions began to regress spontaneously within one month.
Somboonviboon (31)	Man, 35 years old	10 weeks after COVID-19	Bilaterally enlarged hilar, paratracheal, and subcarinal lymph nodes revealed by CT. Panuveitis and papillitis found during an eye examination	One-month of prednisolone therapy resulted in reduced intrathoracic lymph nodes, improved vision, and reduced hyperemia of the optic nerve head.
Capaccione (32)	Man, 61 years old	14 months after severe COVID-19	Lymphadenopathy of mediastinal and intrathoracic lymph nodes	Systemic prednisolone therapy
Rodrigues (33)	Woman, 57 years old	After COVID-19	Erythematous, symmetrical, non-pruritic papules and plaques	Systemically administered prednisolone together with Azathioprine. Clinical improvement.
Palones (34)	Woman, 45 years old	2 weeks after COVID-19 onset	Cough and 2–3 cm erythematous skin rashes, painful on palpation. CT showed lung infiltrates with granulomatous changes.	Inhaled corticosteroids: six-month follow-up X-ray improvement
Rabufetti (35)	Man, 31 years old	2 weeks after COVID-19 onset	Erythematous skin lesions and mediastinal lymphadenopathy found by CT	Systemically administered prednisolone: regression of skin lesions
Pokhriyal (36)	Man, 64 years old	1 week after COVID-19 onset, using PCR (+)	Shortness of breath and symptoms of pneumonia concomitant with a large 6.3 × 4.7 cm size lung mass detected in the right upper lobe along with enlarged bilateral lymph nodes with signs of granulomatous inflammation	Simultaneously administered inhaled corticosteroids together with systemic prednisolone therapy: clinical and X-ray improvement.

Hence, patients of different sexes and ages during or 2–3 weeks after the onset of COVID-19 had various manifestations of sarcoidosis, ranging from cutaneous erythematous manifestations to pulmonary infiltrative changes. For example, a 72-year-old woman was found to have cutaneous painful, firm nodules representing noncaseating sarcoid-like granulomas, presented 2 weeks after recovery from COVID-19 pneumonia (29).

Another female patient was found to have swelling over old scars and *de novo* papules and vesicles 1 month after being diagnosed with COVID-19. Dense subcutaneous nodules appeared on the elbows. A needle biopsy obtained from an old scar-infiltrated plaque revealed during histopathological examination non-necrotic exposed granulomas in the superficial and deep dermis, suggestive of sarcoid granuloma. Similar data were obtained after an excisional biopsy of the subcutaneous nodule. However, no SARS-CoV-2 RNA was detected in the affected areas (30). Similar symmetrical erythematous non-pruritic papules and plaques were observed in a 57-year-old COVID-19 convalescent woman who was found to have sarcoid granulomas after histological examination of a skin biopsy (33).

Some patients were noted to have bilateral lymphadenopathy affecting hilar, paratracheal, and subcarinal lymph nodes based on chest CT scans performed 10 weeks after COVID-19. At the same time, panuveitis and papillitis were simultaneously found. Histological examination of transbronchial biopsy samples from intrathoracic lymph nodes revealed pathological sarcoid granulomas (31). Such data were recapitulated when examining the patient 14 months after severe COVID-19 (32). The development of cardiac sarcoidosis after acute COVID-19 has been rarely described (37). One of the recently published cases described an infiltrative process based on respiratory CT data paralleled with a severe cough. The patient was also found to have painful 2–3 cm cutaneous erythematous changes after COVID-19 infection (34). In addition, histochemical examination of a similar clinical case revealed a large number of specifically stained CD4+ T cells along the periphery of the granuloma (35).

Thus, it can be concluded that SARS-CoV-2 may be one of the cues resulting in the development of sarcoidosis-related inflammatory changes, ranging from affected skin to lymphadenopathy and pulmonary infiltrative foci, which may be paralleled by profoundly altered T and B cell immune responses.

## 4 B cell subset alteration in sarcoidosis during COVID-19

The phenotypic profile of B cells may indirectly mirror the functions of some B cell subsets and, therefore, their relevant role in the pathogenesis of sarcoidosis and COVID-19.

The role of the adaptive humoral arm in the pathogenesis of sarcoidosis is additionally evidenced by data showing that one of the signs of this disease is coupled to polyclonal hypergammaglobulinemia (38). Despite this, several reports noted that patients with sarcoidosis have normal serum immunoglobulin levels (39), but molecular biological analysis of IgA and IgG transcripts revealed a high frequency of somatic hypermutations suggesting persistent antigenic B cell stimulation (40). In addition, the latter study using histological methods showed B cell accumulation in pulmonary foci, which agrees with previous observations (41).

Recent studies described that patients with sarcoidosis had reduced levels of peripheral blood “naïve” IgD + CD38– and memory IgD–CD38+ and IgD–CD38– B cell subsets, while activated IgD + CD38+ and IgD + CD38++ were increased (42). This may be due to the migration of such cells to the lymph nodes and, possibly, to the foci of inflammation (43). Further investigation allowed to identify that the peripheral blood memory B cell population was altered in sarcoidosis patients compared to healthy subjects due to the decreased levels of “unswitched” (IgD + CD27+) and “class-switched” (IgD − CD27+) memory B cells, whereas the levels of CD19 + CD24+++CD38+++ and CD19 + CD5 + CD27– regulatory B cells were increased (42). The latter B cell phenotype is characterized by a stronger anti-inflammatory potential due to IL-10 production (44). Saussine et al. also found a higher count of IL-10-producing regulatory B cells in the peripheral blood of active chronic sarcoidosis patients (43). Classically, it is recognized that the CD5+ B cells could be found in various human tissues being capable of autoantibody production (including rheumatoid factor and anti-ssDNA antibodies) and that the count of CD5+ B cells is expanded in autoimmune diseases such as rheumatoid arthritis and Sjögren’s syndrome (45, 46). Unfortunately, little is known about the functional potential of CD5+ B cells and the role they may play in the pathophysiologic mechanisms of human autoimmune diseases. In mouse models, CD5+ B cells have been shown to belong to a B1a subset that is typically located in the peritoneum and produces low-affinity autoreactive IgM antibodies (47). Furthermore, IL-10 is produced by CD5+ B cells and is involved in the regulation of autoimmunity during experimental autoimmune encephalomyelitis in mice (48). In humans, CD5 expression could be found on the cell surface of CD24+++CD38++ T1 transitional B cells (49), but recent data suggest that these cells are capable of producing low levels of IL-10 compared with other transitional B cell subsets (50). Intriguingly, some reports show that CD5 can be considered an activation marker for human B cells. Hence, human CD5-negative B cells may be suggestive of an *in vitro* activated population after exposure to phorbol-myristic acetate (PMA) or EL4 thymoma cells, which turn into CD5-positive cells (51). Finally, human CD5+ B cells have not been clearly characterized, although they may act as additional diagnostic and therapeutic targets in several autoimmune diseases.

In addition, high-frequency CD19+/–CD20–CD27++ plasmablasts in peripheral blood have been demonstrated in sarcoidosis (52). A special role in the development of inflammation has been attributed to IgA+ plasmablasts, which are found in substantial numbers in the peribronchial infiltrates of sarcoidosis patients (53). To assess B cell activation, serum B cell activating factor (BAFF), belonging to the TNF family, was also analyzed and found to be elevated in sarcoidosis, paralleling the activated inflammatory process (43, 54). BAFF levels were correlated with severe disease course and clinical manifestations (55). Moreover, elevated vitreous BAFF levels have been also noted in patients with sarcoidosis uveitis (56, 57) and granulomas in skin lesions of sarcoidosis (58). Hence, activated B cells are involved in granuloma formation and contribute to the development of not only systemic but also local inflammatory events.

Thus, B cells in sarcoidosis predominantly bear an “activated” phenotype, likely as a compensatory response due to the hyperactivation of pro-inflammatory immune cells and their migration into affected tissues. An increased count of CD19 + CD5 + CD27– cells in bronchoalveolar lavage has been previously reported in sarcoidosis (59), thereby confirming the assumption that Breg cells counteract to suppress inflammatory reactions. However, Breg cells may also contribute to disease progression. In this regard, Mengmeng et al. uncovered an elevated CD19 + CD24 + CD38+ Breg cell count in peripheral blood in active sarcoidosis. Moreover, the level of peripheral blood IL-35-producing Bregs was elevated and correlated with disease activity, while anti-IL-35 antibodies provided better control of sarcoid granuloma development in mice (60). Hence, Breg function in sarcoidosis remains underinvestigated, while recent studies suggest an ambiguous role such cells may play in disease pathogenesis.

B cells also play an important role in the pathogenesis of COVID-19 (61–63). The quality of the B cell response, including the development of high-affinity antibodies and memory B cells, is necessary to prevent the spread of infection and rapid virus elimination (64). In this regard, patients with COVID-19 were found to have decreased peripheral blood “naive” B cell levels along with elevated plasmablast counts (65). Moreover, Kudryavtsev et al. discovered a decline in peripheral blood memory B cell levels along with increased plasmablast counts during acute vs. convalescent COVID-19 in healthy volunteers [I. V. (66)]. Further investigation allowed to find that the peripheral blood levels of “class-switched” (IgD-CD27+) and “unswitched” (IgD + CD27+) memory B cells were significantly reduced during acute disease, while the percentage of double-negative (DN) memory B cells (CD27-IgD-) was markedly increased (67).

Based on the surface expression of CD21 and CD11c, DN memory B cells can be divided into four subsets: DN1 (CD21 + CD11c–), DN2 (CD21–CD11c+), DN3 (CD21–CD11c–), and DN4 (CD21 + CD11c+). DN1 B cells (CD21 + CD11c-) were significantly reduced in severe and critical cases, but not in mild/moderate ones, compared with healthy subjects; DN2 B cells (CD21–CD11c+) were significantly increased in severe, mild/moderate, and critical cases; the DN3 (CD21–CD11c–) subset increased along with escalating disease severity; and the DN4 subset was not determined in peripheral blood samples from either COVID-19 or healthy subjects (68, 69). In COVID-19, the DN3 B cell subset may migrate to the site of inflammation to further contribute to developing formation (68). Moreover, this cell type can also contact CD4+ T cells in the lungs, thereby accounting for local antibody production (68). The presence of the DN3 subset may be related to a dominant extrafollicular B cell response, implying earlier production of antibodies with low-affinity maturation, a lack of memory cell formation, or the emergence of “short-lived memory B cells” unable to produce high-affinity antibodies upon repeated antigen encounters (64, 69). Indeed, it has been shown that peripheral blood IgM-positive DN2 and DN3 B cell subsets dominate in patients with severe COVID-19 (70). Moreover, some studies have reported high levels of “extra-follicular” B cell development and low efficiency of somatic mutagenesis during B cell maturation in peripheral lymphoid organs (71, 72), which may also be paralleled with the emergence of low affinity antibodies that can, among other effects, elicit autoreactivity. A recent study demonstrated a positive correlation between peripheral blood DN3 B cell subset levels and autoreactive antibodies such as anticardiolipin IgG, antichromatin IgG, and Smith antigen IgG in COVID-19 patients (70). In addition, the increased count of peripheral blood DN B cells in COVID-19 may suggest the prevalence of the “extra-follicular” pathway for B cell development during the antigen-specific humoral response that may be predominant in the severe disease course (73, 74). Furthermore, the prevalence of “extra-follicular” mechanisms in antigen-dependent B cell maturation has been observed in a typical autoimmune disease such as systemic lupus erythematosus (75, 76).

Such alterations in developing B cells are also suggested by the high serum BAFF levels observed in severe acute COVID-19 (77, 78). COVID-19 non-survivors vs. survivors demonstrated higher serum BAFF levels (79). Moreover, upregulated lung BAFF expression was observed in COVID-19 infection (80), while Alturaiki et al. found higher blood BAFF levels in mild SARS-CoV-2 (81). Elevated BAFF levels may underlie the maintenance of autoreactive B cell clones because this cytokine promotes the survival of CD38high B cells (82, 83), which are able to secrete autoantibodies, therefore suggesting the risk of developing autoimmune pathologies during the long COVID-19 syndrome. On the other hand, anti-BAFF monoclonal antibodies can be effective in the treatment of autoimmune pathologies (84), which could be a crucial component in lowering the risk of developing autoimmune reactions in severe COVID-19 cases.

B cell hyperactivation was also observed after COVID-19 recovery, which can be considered part of the post-COVID-19 syndrome, resulting in autoimmune reactions. It is known that both the percentage and the absolute count of activated B cells bearing a CD19 + CD80 + /CD86 + phenotype remain at a high level after recovery (85). Moreover, COVID-19 convalescents have also been observed to have high peripheral blood levels of upregulated surface PD1-positive plasmablasts and plasma cells (85). In addition to high CD86 expression on B cells, Castleman et al. (86) found high levels of the activation molecule CD69 on CD19+ B cells in COVID-19 convalescents, who were also noted to have a greater percentage of memory B cells compared to acute COVID-19 [I. V. (66)]. However, such cells may be involved in autoreactive effects post-infection, as was found by Vijayakumar et al., who showed that patients who experienced acute respiratory distress syndrome (ARDS) as part of the post-COVID-19 respiratory syndrome had an increased number of airway IgD–CD27+ memory B cells that correlated with CT scan-detected respiratory abnormalities (87). Overall, there is evidence of impaired B cell function after SARS-CoV-2 infection and aberrant expression of major B cell markers, e.g., in patients after severe COVID-19 who experienced downregulated B cell CD19 expression 1 year later (88), which may affect B cell activation upon antigen recognition. Furthermore, after severe COVID-19, patients were noted to lack surface CD21 (involved in the transduction of the surface B cell receptor-linked activating signal) typical of activated self-reactive B cells (86). The levels of the surface inhibitory molecules CD22 and CD72 on B cells were also found to be downregulated. BCR-linked stimulation of B cells with a similar phenotype resulted in hyperactivation of BCR-coupled effector molecules such as pSYK, pBLNK, and pPLCγ2 (86).

## 5 Autoantibodies in sarcoidosis and COVID-19

As mentioned above, the adaptive humoral arm is of particular importance in the pathogenesis of sarcoidosis. In addition to antibody production, some B cell subsets may also play a regulatory role by suppressing activated immune cells through the secretion of anti-inflammatory cytokines such as IL-10 and IL-35 (89).

Recent studies assessing the autoantibody profile demonstrate that class M (IgM) and class G (IgG) immunoglobulins are produced (90), which are able to specifically recognize proteins expressed in different human tissues targeted by sarcoidosis. For instance, uveitis in sarcoidosis patients was noted to be associated with elevated levels of anti-retinal autoantibodies (91). Caforio et al. found that cardiomyocyte-specific and anti-intercalated disc autoantibodies are produced in cardiac sarcoidosis (92). Hence, such findings were mainly observed in cardiac sarcoidosis (92). In addition, other studies have reported autoantibodies against cytoskeletal components and lysosomal trafficking proteins, regardless of the organ affected (93). Moreover, patients with sarcoidosis often had high antinuclear antibody (ANA) titers (58), anti-dsDNA antibodies (94), and those recognizing cyclic citrullinated peptides (95). Analyzing samples collected from 154 sarcoidosis patients uncovered elevated levels of anti-mitochondrial antibody-M2, anti-Ro52, anti-Ro60, anti-SSB, anti-P0, anti-CCP, anti-β2-GP, anti-Sm antibodies, and rheumatoid factor (RF) (96). Bronchoalveolar lavage fluid and sera from patients with sarcoidosis have been found to contain large amounts of IgG capable of specifically recognizing a wide range of different autoantigens (97).

Recently, the theory describing the development of autoantibodies against vimentin has become widespread. Bagavant et al. revealed an increased anti-vimentin IgG titer in patients with sarcoidosis vs. healthy subjects (98). However, despite the discovery of anti-vimentin autoantibodies, other studies refuted that they could have a profound impact on the overall pathogenesis of the disease (99). Therefore, the question regarding the role of autoantibodies in the pathogenesis of sarcoidosis remains open. In this context, an experimental model of granulomatous inflammation has been proposed after inoculating mice with vimentin-rich patient-derived blood samples (98).

Both during and after acute COVID-19, phenotypical and functional impairment of B cells was noted when they acquired the potential to produce autoreactive antibodies. B cells are mainly known to produce antibodies as a determining arm of adaptive humoral immunity in both infectious events and autoinflammation. There are emerging data regarding the activation of autoreactive B cell clones in patients with COVID-19 (100). Moreover, COVID-19 convalescents were noted to contain peripheral blood autoantibodies against various cytokines (IFN-α, IFN-ω, IFN-γ, IL-1β, IL-6, IL-10, IL-17, IL-21, and GM-CSF) and chemokines (CCL2, CXCL1, CXCL7, CXCL13, and CXCL16) (101, 102). In addition, elevated titers of autoantibodies specific for chromatin, cardiolipin, and Smith antigen have been observed (86). The latter was found in systemic lupus erythematosus (SLE) (103). At the same time, Chang et al. uncovered anti-cytokine autoantibodies in acute COVID-19 [S. E. (104)], which were found in approximately 50% of 147 COVID-19 convalescent patients. Another study showed that 101 out of 987 patients during acute SARS-CoV-2 infection had type I interferon-neutralizing antibodies, including those targeting IFN-ω (13 patients), 13 different types of IFN-α (36 patients), or both (52 patients) (105). It should be noted that the appearance of anti-type I IFN antibodies has also been observed in classic autoimmune diseases such as systemic lupus erythematosus (106) and systemic sclerosis (107). Furthermore, that study also identified three patients bearing blocking antibodies against IL-6, IL-22, and IL-12p70 (105). Another study obtained similar data (108), demonstrating that COVID-19 patients had elevated levels of autoantibodies recognizing various immunomodulating cues, including cytokines, chemokines, complement components, and cell surface proteins. Hence, it has been suggested that such autoantibodies interfere with the effective functioning of the human immune system and affect immune control of viral infection by inhibiting cell-to-cell signaling and altering the composition of circulating immune cells, ultimately exacerbating the severity of SARS-CoV-2 infection.

Interestingly, Woodruff et al. showed that virtually no antibodies against Sm/RNP, Ro, La, and dsDNA were detected in severe COVID-19 cases, whereas antinuclear antibodies were detected in approximately 40% of these patients (109). Moreover, approximately 40% of COVID-19 patients had antibodies against carbamylated vimentin (anti-CarP), which plays an important role in the destruction of connective tissue in SLE and rheumatoid arthritis (110, 111).

On the other hand, the majority of patients with ARDS were found to have elevated antinuclear antibody levels (112), which raises the question of the existence of similar mechanisms underlying lung damage in SARS-CoV-2 infection and exacerbation of some autoimmune diseases, including SLE and rheumatoid arthritis (113). Similar data have been obtained by several independent groups describing elevated antinuclear antibody (ANA) and antineutrophil cytoplasmic antibody (ANCA) titers after acute COVID-19 (112, 114, 115). Moreover, ANAs may be closely related to hair loss as a part of the long COVID-19 syndrome (116). On the other hand, detected rheumatoid factor may be another example of emerging autoantibodies after COVID-19, whose increase during acute disease was closely associated with its severe and critical course (117). Other studies described anti-platelet autoantibodies as able to markedly impact the severity of acute coronavirus disease (118). Gagiannis et al. also observed an elevated titer of anti-PM-Scl−/anti-Scl-70 antibodies in patients who developed pulmonary fibrosis, which raises the question of long-term consequences related to severe COVID-19. In addition, the detection of increased autoantibody titers specific to phospholipids (anticardiolipin, anti-β2-glycoprotein I, anti-phosphatidylserine/ prothrombin) in acute COVID-19 has also been reported (119–121).

Furthermore, anti-cytokine autoantibodies associated with human inborn genetic defects mimic primary immunodeficiencies. Such pathologies are called “phenocopies of inborn errors of immunity” (122) and are often found in adults (123). Due to the appearance of autoantibodies specific for various kinds of cytokines, such as autoantibodies against type I IFN, IFNγ, GM-CSF, IL-17A, IL-17F, IL-22, IL-23, and IL-6, patients are predisposed to various infectious diseases, as well as bacterial, fungal, or viral infections (124). Patients with inborn defects in the IFNAR1 and IFNAR2 genes encoding type I IFN have a severe course of acute respiratory viral infections such as influenza and COVID-19 (105, 124). Neutralizing autoantibodies related to SARS-CoV-2 or phenocopy of inborn errors of immunity leads to severe infection and contributes to autoimmune disease.

An autoantibody spectrum detected in sarcoidosis and COVID-19 is shown in Table 2.

**Table 2:** Autoantibody spectrum in sarcoidosis and COVID-19.

Autoantibody type	COVID-19	Sarcoidosis	Functions
Antinuclear antibodies (ANA), including anti-dsDNA antibodies, anti-Ro52 antibodies, anti-Ro60 antibodies, anti-SSB antibodies, anti-P0 antibodies, anti-chromatin antibodies, anti-Sm antibodies, anti-PM-Scl−/anti-Scl-70 antibodies	↑ Basic-Jukic et al. (114), Castleman et al. (86), Gagiannis et al. (112), Manav et al. ((116), Pascolini, et al. (115), and Woodruff (109))	↑ Shi et al. (96), Ueda-Hayakawa (58), and Weinberg et al. (94)	Destroy cell nuclear material; immune complex-mediated damaging effect on host tissues
Anti-cyclic citrullinated peptide antibodies (anti-CCP)	↑ Woodruff (109)	↑ Kobak et al. (95) and Shi et al. (96)	Induce bone erosion via osteoclast activation
Anti-mitochondrial antibody-M2, anti-ribosomal-P0-antibodies	Not significant Woodruff (109)	↑ Shi et al. (96)	Directed against lipoproteins on the inner mitochondrial membrane and ribosomes; contribute to biliary cirrhosis and SLE, respectively
Rheumatoid factor (RF)	↑ Jeong et al. (117)	↑ Shi et al. (96)	Contribute to chronic inflammation of the synovial membrane; promote cartilage destruction
Anti-vimentin antibodies	↑ Woodruff et al. (109)	↑ Bagavant et al. (98) and Hanoudi et al. (93)	Disorganization of cytoskeleton components
Antineutrophil cytoplasmic antibodies (ANCAs)	↑ Basic-Jukic et al. (114)		Attack neutrophils, leading to their degranulation and destruction
Antiphospholipid antibodies (Anticardiolipin, anti–β2-glycoprotein I, anti-phosphatidylserine/prothrombin)	↑ Castleman et al. (86), Xiao et al. (119), Zhang et al. (120), and Zuo et al. (121)	↑ Shi et al. (96)	Damage to the endothelium and hemostatic system; contributes to thrombosis
Anti-cytokine autoantibodies, including anti-type I IFN antibodies	↑ Acosta-Ampudia et al. (101), Bastard et al. (105), Chang et al. (104), Garmendia et al. (102), and Wang et al. (108)		Block cytokine signaling pathways, leading to the spread of infectious agents
Organ-specific autoantibodies (anti-retinal autoantibodies, anti-glomerular basement membrane (GBM) antibodies, anti-cardiomyocyte antibodies)	↑ Woodruff et al. (109)	↑ Avendaño-Monje et al. ((91) and Caforio et al. (92))	Damage to various tissues

## 6 Alterations in Th subsets in patients with sarcoidosis and acute COVID-19.

It should be noted that the pathogenesis of sarcoidosis has long been associated with Th1 cell hyperactivation (125); sometime later, the key role in its development was considered to be due to altered Th1/Th17 cell ratios in the foci of granuloma formation (126). Currently, it is also common to pay attention to Th2 cells as a potential player in granuloma formation (127). This assumption is confirmed by clinical observations pointing to increased levels of peripheral blood CCR4 + CD4+ cells in sarcoidosis, as well as elevated concentrations of CCL17 chemokines both in the blood serum and at the site of granuloma formation (128). Moreover, underlying *in vivo* experimental models of pulmonary fibrosis demonstrated the key role of CCR4 ligands (primarily CCL17, but also CCL22) in tissue fibrosis, where blockade of CCL17 effects in mice resulted in lesion reduction (129). Excessive Th2 cell activation in patients with sarcoidosis is also confirmed by upregulated expression levels of IL-13 mRNA (one of the key Th2 cytokines) in peripheral blood mononuclear cells (130). Moreover, animal models (131) and tissue specimens obtained from patients with sarcoidosis (132) showed that hyperproduction of Th2 cytokines is accompanied by tissue macrophage activation and differentiation toward the M2 phenotype, which contributes to the development and maintenance of tissue foci of chronic inflammation, the formation of granulomas, and foci of fibrosis.

Apart from analyzing the balance between Th1 and Th2 cells, studies investigating the pathogenesis of sarcoidosis have paid special attention to the role of Th17 cells and their specific subpopulations. Data on temporal changes in peripheral blood Th17 cells are very conflicting, because some studies indicate an increased level of CCR6+ effector T helper cells (CD45RA-CD45R0+) in patients vs. controls (133), whereas others evidence that, for example, the level of IL-17A-producing cells in patients’ peripheral blood was markedly below the control range (134). We found no significant differences in the level of CCR6-expressing Th cells not only between acute or chronic onset sarcoidosis but also when compared with the control group. At the same time, elevated blood serum levels of cytokines and chemokines such as IL-17, IL-22, IFN-γ, and CCL20 produced by patient Th17 cells are noted in the majority of studies (135). Subsequently, higher levels of these cytokines have been shown to be contained not only in bronchoalveolar lavage fluid (BALF) and granulomatous tissue but also in the cell types involved in their production (136). In parallel, the discovery of a fundamentally novel T helper cell population, T helper 17 (Th17) cells, in addition to a unique highly specialized subtype, Th17.1 cells, capable of producing both Th1 and Th17 cytokines, including IFNγ and IL-17A, suggested that sarcoidosis might be autoimmune in nature (137, 138). A series of studies have detected higher levels of Th17.1 cells and related proinflammatory cytokines in the peripheral blood and BAL fluid in sarcoidosis (133, 139). Moreover, the level of total CCR6+ Th cells, including Th17.1 cells, was significantly increased in lung-related lymph nodes of patients compared to controls (140).

Regarding the pathogenesis of COVID-19, the role of the Th1 cell subset, which plays a key role in the immune response against intracellular pathogens, is quite controversial. For instance, some studies suggest that IFNγ-producing Th1 cells may play a positive role in COVID-19, and their higher activity may be associated with a milder disease course (141, 142). On the other hand, older patients, who are usually characterized by severe COVID-19, were noted to have decreased levels of IFNγ-producing virus-specific cells, which also indirectly indicates an important role of Th1 cells in the development of an effective immune response (143). However, Th1 cell-induced IFNγ and TNFa overproduction in response to SARS-CoV-2 infection, as well as the massive death of virus-infected cells, can result in damaged lung tissue and trigger acute respiratory distress syndrome. In particular, during acute COVID-19 infection, the migration of Th1 cells into inflamed tissues has been indirectly evidenced by their slightly decreased percentage in the peripheral blood, which has been observed in several studies (144–146). However, some studies have revealed an accumulation of “atypical” peripheral blood Th1 cells expressing surface markers such as CD161 and IL-1RI (146), which are more typical of Th17 cells (or “non-classical” Th17.1 cells) in patients with severe COVID-19 pneumonia. Th2 cells primarily target multicellular pathogens, but virus-specific Th2 cells are detected in COVID-19 (147), while high levels of Th2 cell cytokines are found in the blood serum of patients during the acute phase of the infection (144). Patients also had an increased percentage of peripheral blood T helper cells expressing surface CCR4 along with nuclear GATA3 (148). A rise in peripheral blood Th2 cells bearing the CXCR3–CCR6– phenotype was closely related to unfavorable outcomes in severe COVID-19, which allowed for it to be considered as an independent prognostic marker (149). Elevated peripheral blood Th2 cell levels and hyperactivation may be closely related to associated symptoms such as intestinal hypermotility, gastric acidification, and dyspnea, which accordingly could be considered typical defense mechanisms to remove parasites via Th2 cytokines (150). With regard to inflamed lung tissues, BALF cells obtained from patients with severe COVID-19 were shown to have upregulated expression of not only the genes encoding crucial cues accounting for Th2 cell “polarization” (*GATA3*, *IL4R*, and *MAF*) but also did not differ in production levels of key Th2 cytokines when assessing patients with varying degrees of COVID-19 severity (151). Moreover, COVID-19 convalescent patients were found to have additionally high peripheral blood Th2 cell levels that persisted for several months, while the concentrations of IL-4, IL-5, and IL-13 did not differ significantly from those in the control groups [F. (152)].

Analysis of the Th cell subset profile in COVID-19 revealed a decline in the percentage of Th17.1 and Th1 lymphocytes capable of producing IFN-γ (145). Moreover, T helper cells from SARS-CoV-2-infected patients contained more IL-17A and IL-2 in response to *in vitro* stimulation, compared to healthy volunteers (148). At the same time, the aforementioned work showed a lower percentage of T helper cells bearing surface key Th17 antigens (CD161 and CCR6) whereas the level of Th2-positive (CCR4 and GATA3) cells was significantly elevated compared to the control group. Similar data were obtained using molecular biology methods, which revealed that peripheral blood CD4+ T cells from patients with severe COVID-19 had downmodulated expression of Th17-associated genes, e.g., *RORC*, *IL17A*, *IL17F*, and *CCR6* (151). However, another study showed that in the peripheral blood specimens of COVID-19 patients, the percentage of Th17 and follicular T cells was higher, paralleled by moderately decreased Th1 cell levels, whereas that for Th2 and Th17.1 cell subsets did not differ compared with the control group (146).

It is possible that Th17 cells migrate to the site of inflammation with varying efficiency at different stages of the infection process. This explains why the BALF data are so important because they point to an accumulation of Th17 cells bearing a “pro-inflammatory” phenotype in the affected lung tissue (153). For instance, lung tissue specimens obtained from COVID-19 patients were enriched for cells co-expressing CCR6 and IL17A and also had high levels of IL-6, IL-17A, GM-CSF, and IFNγ found in BALF. The crucial role of Th17 cells in the pathogenesis of COVID-19 is suggested by the data showing that after successful completion of the infection process and pathogen elimination, memory Th17 cells remain persistently in circulation. In this regard, some studies have uncovered the emergence of virus-specific memory Th17 cells capable of producing IL-17A, IL-17F, and IL-22 in response to *in vitro* stimulation with the SARS-CoV-2 S protein-derived peptide pool (147).

Thus, the pathogenesis of sarcoidosis and COVID-19 is closely related to profound alterations in the major T helper cell profile coupled to the regulation of the three types of inflammatory reactions, such as the type 1 response aimed at eliminating intracellular pathogens, the type 2 response associated with control over multicellular pathogens and relevant toxins, and the type 3 response necessary for effective elimination of pathogens (bacteria and fungi) localized in the intercellular space of various host tissues. These alterations may directly impact the activation of the humoral immune response, which is controlled by follicular T helper cells.

## 7 Follicular helper T cell subset alterations

Cross-talk between follicular helper T cells (Tfh) and B cells at the border of T cell and B cell areas in the lymphoid follicle is necessary for the development of an effective humoral immune response (154). Tfh cells are characterized by surface expression of the chemokine receptor CXCR5, which is necessary for migration to the B cell zone (154). Tfh plays a pivotal role in B cell maturation and differentiation during the germinal center reaction that occurs in peripheral lymphoid organs (155–157). Tfh cells also control antibody class switching in B cell-produced immunoglobulins, eliciting somatic hypermutation and clonal selection of high-affinity B cells that further differentiate into plasma cells and memory B cells. Thus, this CD4+ T cell is required for the production of both high-affinity pathogen- and self-antigen-specific antibodies and autoantibodies, respectively. The detection of CD4 + CXCR5+ T cells is crucial in both infectious and autoimmune diseases to assess the quality of the adaptive humoral immune response. At the same time, the half-life of Tfh cells in peripheral blood is highly heterogeneous. For instance, circulating Tfh cells can be divided into four major subsets based on CXCR3 and CCR6 coexpression: CXCR3 + CCR6 − Tfh1, CXCR3 − CCR6 − Tfh2, CXCR3 − CCR6+ Tfh17, and CXCR3 + CCR6+ Tfh17.1 cells. Moreover, these cell types mimic Th1, Th2, classic Th17, and pro-inflammatory Th17.1 cells, respectively, in terms of functional activity and phenotype (158–160). Altered Tfh cell function, as well as an altered balance between their individual circulating subsets, is closely related to pathological effects on the activity of the overall antigen-specific humoral immunity. This may potentially account for the fact that an imbalance between the proportion of Tfh1 cells, on the one hand, and Tfh2 cells, along with Tfh17 cells, on the other hand, is observed in rheumatoid arthritis, SLE, Sjogren’s syndrome, multiple sclerosis, type 1 diabetes mellitus, and other conditions (161).

Regarding sarcoidosis, the profile of Tfh cells has only recently been investigated. In particular, Kudryavtsev et al. assessed Tfh subset composition by assessing the expression of differentiation molecules and chemokine receptors (42). It was revealed that in chronic sarcoidosis, upregulated CXCR5 expression was observed on central memory CCR7 + CD45RA– CD4+ Т cells along with an elevated percentage of peripheral blood CXCR3–CCR6– Tfh2-like cells. While evaluating the surface chemokine receptor expression on central memory Tfh cells, it was found that in pulmonary sarcoidosis peripheral blood samples contained an elevated percentage of Tfh2 (CXCR3 − CCR6 − CCR4+), Tfh17 (CXCR3 − CCR6 + CCR4+), and dual-positive Tfh17 (CXCR3 + CCR6 + CCR4+) paralleled with significantly reduced Tfh1 (CXCR3 + CCR6 − CCR4−) and Tfh17.1 (CXCR3 + CCR6 + CCR4−) levels [I. (42)]. A decline in peripheral blood Tfh1 and Tfh17.1 cell levels in sarcoidosis seems to be related to their migration to organs and tissues affected by sarcoid granulomas. Zhou et al. (9) also noted an altered Tfh cell subset composition associated with increased levels of Tfh2 and Tfh17 cells but decreased levels of Tfh1 and Tfh17.1 cells. Notably, Tfh2 and Tfh17 cells contributed to the survival of activated “naive” B cells, their transformation into plasma cells, and antibody class switching, whereas Tfh1 cells performed regulatory functions related to the emerging humoral immune response (159). It is possible that an imbalance between pro- and anti-inflammatory Tfh cell subsets may play a paramount role in abrogating the overall development of autoimmune reactions.

Indeed, other studies have reported elevated bronchoalveolar lavage fluid (BALF) levels of CXCR3 chemokine receptor-expressing follicular T cells (53) found on both Tfh1 and Tfh17.1 cells. Furthermore, d’Alessandro et al. (162) showed upregulated expression of the integrin molecule CD103 on peripheral blood, BALF, and intrathoracic lymph node biopsy specimens in patients with sarcoidosis, indirectly confirming a theory of migration of such cells into inflammatory foci. Ly et al. verified the presence of CD4 + CXCR5+ T cells in sarcoidosis-related skin lesions (163). However, other reports, on the contrary, revealed an increased level of peripheral blood Tfh1 cells along with decreased levels of BALF Tfh1 and Tfh2 cells in chronic sarcoidosis (59). Hence, ambiguous data regarding the role of follicular T cells and B cells in this pathology implies a need for their more thorough study.

Follicular T helper cells are responsible for the quality of the antiviral B cell response in COVID-19, owing to the fact that Tfh cell-mediated stimulation of B-lymphocytes enables more fine-tuned differentiation, resulting in the emergence of long-lived memory B cells and plasmacytes that produce virus-specific high-affinity antibodies (64). In particular, circulating Tfh cells from COVID-19 patients support *in vitro* B-cell differentiation into antibody-secreting plasma cells and antibody production, whereas altered differentiation of SARS-CoV-2-specific Tfh cells at early stages of infection was closely related to severe COVID-19, accompanied by delayed production of high-affinity antibodies and disease progression (164). Subsequently, the level of SARS-CoV-2-specific cTfh cells in COVID-19 convalescent patients correlated with SARS-CoV-2-neutralizing antibody titers, which evidenced a crucial function of this cell type in maintaining protective immunity [J. (165)].

However, data regarding circulating Tfh cell levels in acute COVID-19 as well as in the post-infection period have been rather ambiguous. For instance, one study noted a decline in CD4 + CXCR5+ T cell frequency, particularly in severe disease (144). In contrast, other studies have reported an increase in peripheral blood Tfh cell levels (146), especially for those cell subsets expressing activation markers such as PD-1 (166), HLA-DR and CD38 (67), and ICOS (167). Importantly, such cell types are highlighted by high expression of the proliferation marker Ki67 (67), suggesting the emergence of post-clonal expansion Tfh cells in an overactivated state that have entered the circulation in severe COVID-19. However, upon SARS-CoV-2 infection, Tfh cell differentiation becomes altered (151). By examining lymphoid organ autopsy samples, Kaneko et al. detected atrophy of the lymph node germinal center (B-dependent zones) (73), which may be due to a heightened cytokine storm wherein TNF-α blocks Bcl-6+ Tfh cell differentiation. This may be related to an enhanced local Th1 cell response. It has been shown that Th1/Tfh1 alters the differentiation of follicular T helper cells, promoting antibody production (73, 168). However, even CD4 + CXCR5+ Tfh cells lacking Bcl-6 expression can interact with and induce the proliferation of naive extrafollicular B cells. Thus, patients with acute COVID-19 infection were found to have enhanced functional and proliferative Tfh cell potential, but impaired differentiation negatively affected the B cell immune response. Importantly, such an impaired adaptive humoral response is often observed in severe COVID-19 and testifies to its inability to result in fully-fledged pathogen-specific humoral immunity (73).

When the CD45RA- memory Tfh cell population was subdivided into Tfh1, Tfh2, and Tfh17 subsets based on surface chemokine receptor expression, it was found that acute COVID-19 was associated with a decreased percentage of Tfh1 paralleled with elevated Tfh17 levels (169). Juno et al. unraveled that among all Tfh cell subsets, the SARS-CoV-2-specific antiviral response peaked in Tfh17 cells, whereas Tfh1 and Tfh2 cells were most closely related to blood plasma SARS-CoV-2-specific antibody neutralizing activity (170). COVID-19 convalescent patients continued to carry an altered profile of circulating Tfh cell cells. Although the total Tfh population did not differ between healthy and convalescent subjects, the level of CXCR3-expressing Tfh1 cells was reduced. Interestingly, in a cohort of recovered patients, the percentage of Tfh1 cells that correlated with the antibody-neutralizing activity peaked in those who had a more severe infection (165). On the other hand, high levels of circulating Tfh1 and Tfh2 were paralleled by markedly reduced levels of Tfh17 (152). COVID-19 convalescents vs. healthy subjects showed a higher frequency of circulating effector memory CCR7loPD-1+ Tfh-em cells and a low level of central memory CCR7hiPD-1+ Tfh-cm cells [F. (152)]. In addition, some studies reported enhanced Tfh cell activity in COVID-19 convalescents [F. (152, 171)], which may contribute to the emergence of autoimmune and allergic reactions during post-COVID-19 syndrome. Furthermore, Tfh cell activity depends on the severity of SARS-CoV-2 infection, such that the level of effector memory Tfh-em cells promoting class switching to IgG antibodies remains high for a long time in patients recovering from severe COVID-19 (152). Kudryavtsev et al. found that Tfh1, Tfh2, and Tfh17 levels remained high in COVID-19 convalescent patients compared with healthy subjects (66). Hence, it may serve as a basis for the development of an autoimmune process with altered immune tolerance often observed in post-COVID-19 syndrome.

A comparison of circulating Tfh cell subpopulations in sarcoidosis and COVID-19 is presented in Table 3.

**Table 3:** Patterns of circulating follicular helper T cell fluctuations in sarcoidosis and COVID-19.

Cell subsets	COVID-19	Sarcoidosis	Functions
Tfh1	↑ (Golovkin et al. (169) and Kudryavtsev et al. (66)) ↓ Zhang et al. (165)	↑ d’Alessandro et al. (162) ↓ Zhou and Arce (9)	Suppress Tfh2-dependent antibody induction, regulate humoral immune response
Tfh2	↑ Kudryavtsev et al. (66)	↑ Kudryavtsev et al. (42) and Zhou and Arce (9)	Contribute to the differentiation and proliferation of activated “naive” B cells, primarily eliciting antibody class switching to IgA and IgG
Tfh17	↑ Golovkin et al. (169), Kudryavtsev et al. (66)	↑ Zhou and Arce (9)	Contribute to the differentiation and proliferation of activated “naive” B cells, primarily eliciting antibody class switching to IgE and IgG
Tfr	↓ Søndergaard et al. (172) and Zahran et al. (173)	↑ d’Alessandro et al. (162) and Igor Kudryavtsev et al. (174)	For antigen-specific vs. antigen-nonspecific B cell clones, it promotes the formation and survival of the former along with the inhibition of the latter, suppressing Tfh cells

## 8 Follicular regulatory helper T cells – Crucial players in the antigen-specific humoral response

It is believed that Tfr represents one of the regulatory T cell subsets that may originate from both thymic Tregs via thymic selection and emerging antigen TCR specificity, or from CXCR5 and BCL-6-co-expressing FoxP3+ cells arising *de novo* in peripheral lymphoid organs (175–177). Follicular regulatory helper T cells play a rather ambiguous role in regulating the humoral immune response. On the one hand, they contribute to the survival and proliferation of B cells specific for some environmental foreign antigens, while on the other hand, they are able to suppress the proliferation and differentiation of foreign antigen-nonspecific B cells by creating a proper environment that mainly promotes the development of antigen-specific germinal center B cell clones in peripheral lymphoid organs (175, 178). Moreover, Tfr cells suppresed Tfh activity, leading to a restrained germinal center response at the stage of Tfh cell-mediated B cell costimulation, which appears to be the key function of this Treg population (175). On the other hand, Tfr cells act to suppress not only Tfh but also B cells, resulting in downregulated antibody production (179). It has also been hypothesized that during germinal center formation, Tfrs regulate the production of antigen-specific antibodies during the primary immune response, and with repeated antigen encounters, their role in regulating the humoral response becomes less prominent (180). On the contrary, Tfrs are thought to play a key role in the later stages of the germinal center response (181).

In the context of the development of autoimmune pathologies, a suppressive role of Tfr has been demonstrated, contributing to the limitation of the autoimmune humoral response (179, 180). Potentially, it may explain why a key role in the development of efficiently functioning Tfr cells is related to their thymic differentiation. Analyzing circulating Tfr cells in patients with sarcoidosis showed that within the total CD45RA–CCR7+ central memory Treg population, the proportion of CXCR5+ Tregs was increased, whereas the percentage of thymic CXCR5+ Tregs did not significantly differ from that in the control group (174). Moreover, d’Alessandro et al. (162) showed that the level of peripheral blood CD4highCD25highCXCR5high Tfr cells was increased in sarcoidosis, while the level of alveolar Tfr cells correlated with the Scadding stages. Furthermore, the level of this Tfr cell subset was shown to be higher in bronchoalveolar lavage fluid (BALF) than in peripheral blood in patients with pulmonary sarcoidosis (59). Based on such data, it can be assumed that in sarcoidosis, Tfr functions may be impaired both at the systemic level (e.g., at thymic Tfr differentiation or impaired function in peripheral lymphoid organs) and during their migration to inflammatory foci, where their regulatory functions may also be altered. It is likely that the peripheral blood Tfr subpopulation declines during novel coronavirus infection because Tfr differentiation, acted upon by pro-inflammatory cytokines, is aimed at creating effector Tfh subsets that promote sustained inflammation to eliminate the pathogen in the acute period. Of note, sarcoidosis is a chronic process often accompanied by fibrosis and a marked autoimmune reaction. Therefore, a rise in Tfr cell levels in both peripheral blood and BALF in sarcoidosis may be a compensatory response necessary to curb inflammation at the site of the tissue lesion, where Tfr cell functional activity may not be as effective, which could contribute to pulmonary fibrosis.

Very few clinical cases are available that describe thymoma formation in sarcoidosis. For instance, Hato et al. reported a clinical case in which calcified thymoma and sarcoid granuloma were localized in the lung parenchyma and intrathoracic lymph nodes (182). A clinical case has also been described in which a patient with existing sarcoidosis developed a thymoma (183). In addition, a malignant thymoma has also been reported, and it has been suggested that it provoked the development of autoimmune sarcoid due to impaired T cell tolerance (184). Moreover, Esendagli et al. clearly demonstrated the thymus-related role in the development of sarcoidosis (185). For example, in their reported clinical case, a 53-year-old female patient presented with sarcoid granulomas in the lung parenchyma, intrathoracic lymph nodes, and skin. A thymectomy resulted in the resolution of the sarcoidosis manifestations. In addition, impaired thymic T-cell differentiation in sarcoidosis was also suggested by high expression of non-TCR-mediated cell activation markers in total peripheral blood “naive” Th cells, apoptosis-related proteins, and profoundly dysregulated CD4+ T-cell differentiation (186).

Profoundly altered thymic function and lowered thymic development of various “naive” T cell subsets in acute COVID-19 are evidenced by lowered TREC (T cell receptor excision circles) levels in the peripheral blood in severe and critical COVID-19 (187, 188). Moreover, this may be related to SARS-CoV-2 infection of the thymic epithelial cells, resulting in altered T cell maturation and differentiation (189). Also, impaired thymocyte selection may be accompanied not only by a decreased percentage of functionally active Treg subsets but also with the release of autoreactive T cell clones into the periphery, capable of mounting a response to host self-antigens and eliciting the development of autoimmune pathologies. This may potentially account for a decline in peripheral blood T regulatory follicular cell levels that was noted in acute SARS-CoV-2 infection compared to the control group (172). Moreover, Zahran et al. obtained similar data showing that hospitalized patients with a severe form of COVID-19 had decreased counts of circulating CD4 + CXCR5 + ICOS+Foxp3+ Tfr cells (173). It should be noted that the level of circulating CD45RA–CD127–CD25 + CXCR5hiPD-1hi Tfr also tended to decrease steadily in COVID-19 convalescent patients (152). In addition, a negative correlation between the frequency of circulating Tfr and virus-specific IgM, IgG, and IgA antibodies was observed in the latter cohort of patients. Follicular T regulatory cells may play an important role in controlling the development of the humoral memory response and antibody specificity, as well as interfering with autoantibody formation. Thus, lowered Tfr levels along with increased Tfh levels in acute COVID-19 may contribute to the development of humoral autoimmune reactions and the emergence of autoimmune pathologies during the post-COVID-19 syndrome. While analyzing the role of B cells and diverse Tfh cell subsets in the development of autoimmune events in sarcoidosis along with COVID-19, it is necessary to consider the issue of the disturbed structure of peripheral lymphoid organs, where an interaction between T and B cells occurs and arises under such pathological conditions. For instance, a non-infectious, non-caseating T and B cell-containing granuloma emerges (190, 191). Such lymphocyte cell types mainly reside in the periphery of the granuloma and exert a high proliferative potential as assessed by Ki-67 expression (192). A granuloma *per se* represents a focus of limited granulomatous inflammation primarily involving macrophages, epithelioid cells, lymphocytes, and plasma cells (191). Epithelioid cells may fuse to form giant cells, both along the periphery of the granuloma and in its center (191). A biopsy of intrathoracic lymph nodes collected from patients with chronic sarcoidosis revealed high levels of B cells and follicular T helper cells (162). When comparing B and Tfh cell counts in patients with sarcoidosis, it was found that the level of B cells and CD4 + CXCR5+ T cells was significantly higher in lymph node biopsy compared to BALF and peripheral blood sample (162). Thus, the adaptive humoral response in this pathology plays an important role not only in the systemic but also in the local inflammatory response.

Multiple studies have noted disturbances primarily in the organization of B cell-dependent zones in acute COVID-19, most often being associated with a decreased volume of germinal centers or their full disappearance, apparently resulting in a mild humoral response in patients with severe disease course (73, 193, 194). The number of lymph node follicular dendritic cells, Bcl-6+ Tfh, and B cells is reduced, whereas AID+ B cells, which are usually kept at an intact level (73, 194), are aberrantly located in subcapsular and paracortical lymph node zones (195). On the other hand, extrafollicular plasmablasts, whose development is not tightly controlled by Tfh and Tfr cells, predominantly display an IgM+ phenotype. They can sometimes be found at high levels in the paracortical and medullary zones of lymph nodes (193, 194, 196). However, along with the loss of follicles, some studies reported lymphoid hyperplasia and the mosaic structure of lymphoid tissue (197). Moreover, splenic white pulp was also noted to contain a lowered relative number and volume of lymphoid follicles, and a decreased number of Bcl-6+ B cell-containing germinal centers (73). Hence, severe acute COVID-19 infection was often found to have an inconsistent adaptive immune response due to depletion of the T helper arm and B-dependent zones in the lymphoid organs. However, it is primarily the increased activity of the above cell types that may underlie an allergic or autoimmune pathology in convalescent subjects.

## 9 Conclusion

**Figure 2:**
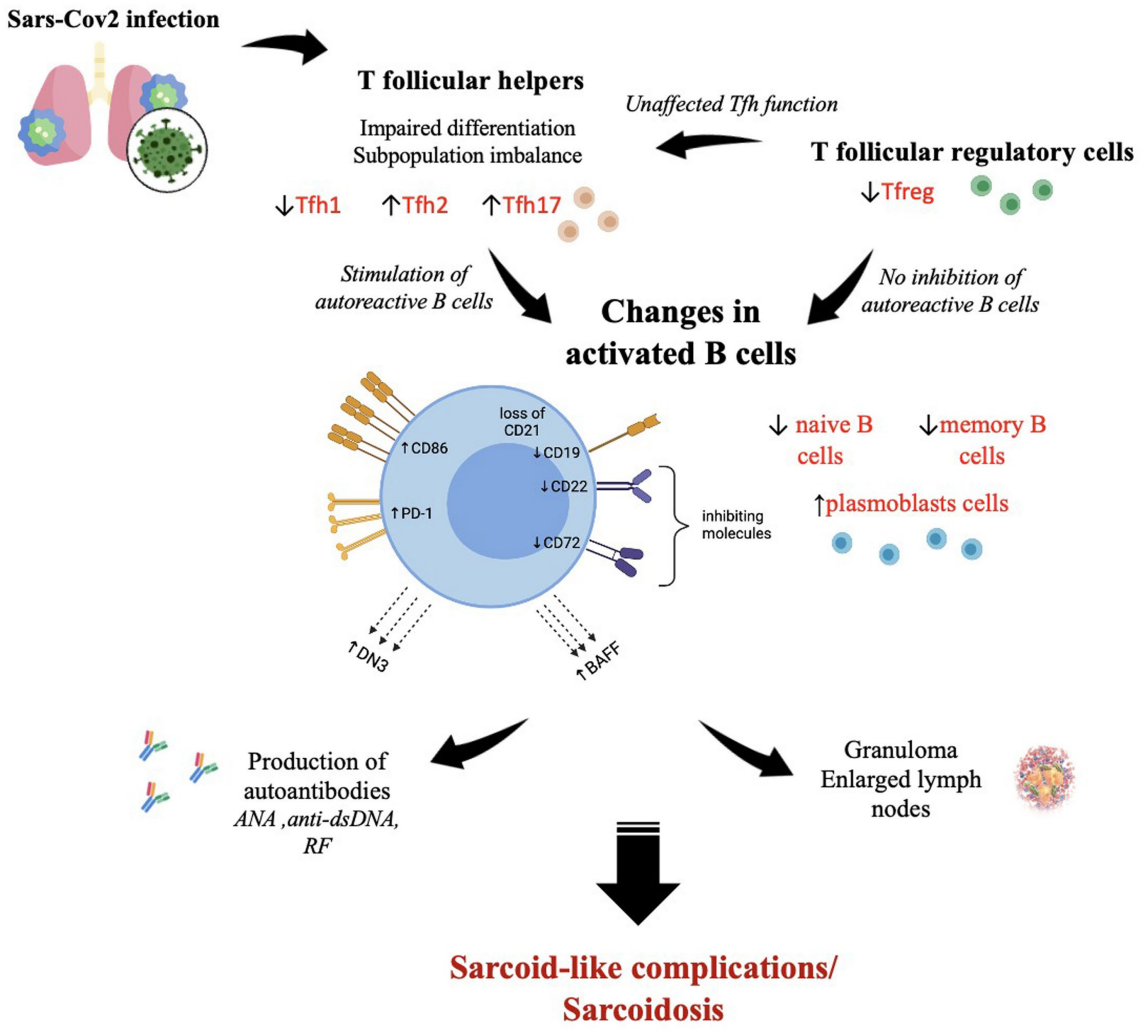
The SARS-CoV-2-induced immunological changes in T follicular helper cells, T follicular regulatory cells, and B cells, which contribute to the development of autoimmune reactions with sarcoid granuloma formation and autoantibody production. ↑—high level; ↓—low level. The figure was drawn by the authors.

Analyzing the available publications allowed us to uncover that SARS-CoV-2 elicits the development of symptoms typical of sarcoidosis a few weeks after COVID-19. The appearance of bilateral lymphadenopathy and eye and skin lesions related to a triggering factor is rather characteristic of an autoimmune process, accompanied by hyperactivation of specific B cell subsets observed after COVID-19, which are also observed in autoimmune inflammation (Figure 2). B cell phenotypic and functional impairments lead to the development of autoreactive potential and autoantibody production. In SARS-CoV-2 infection, follicular helper T cells determine the quality of the virus-specific B cell response. At the same time, Tfh-mediated B cell stimulation may be more finely tuned, resulting in the emergence of long-lived memory B cells and plasmacytes that produce high-affinity antibodies. On the other hand, expanding our understanding of the essential rules of Tfh and Tfr function may allow the development of new approaches to restore the functions and balance between individual subsets of these cell types in the prevention and treatment of autoimmune and inflammatory diseases. The data obtained not only reflect the specific features of sarcoidosis-related autoimmune inflammation associated with SARS-CoV-2 infection but also the need to shed light on the further management strategy of such patients, taking into account the changes identified. At present, the existing concept allows for the monitoring of sarcoidosis patients without therapy. On the other hand, patients with a history of sarcoidosis are required to be prepared for its activation and chronicity. However, the question of establishing immunological criteria accounting for the need for immunotherapy and drug administration remains open.

## Author contributions

AR: Writing – original draft, Conceptualization, Formal analysis. IK: Writing – original draft, Project administration, Writing – review & editing. AM: Writing – original draft. JM: Writing – original draft. DI: Writing – review & editing, Methodology, Writing – original draft. II-S: Formal analysis, Writing – original draft. DK: Funding acquisition, Project administration, Writing – review & editing. AS: Writing – original draft, Project administration.

## Footnotes

^1^ http://www.prisma-statement.org
